# Deep Airway Inflammation and Respiratory Disorders in Nanocomposite Workers

**DOI:** 10.3390/nano8090731

**Published:** 2018-09-16

**Authors:** Daniela Pelclova, Vladimir Zdimal, Martin Komarc, Stepanka Vlckova, Zdenka Fenclova, Jakub Ondracek, Jaroslav Schwarz, Martin Kostejn, Petr Kacer, Stepanka Dvorackova, Alexey Popov, Pavlina Klusackova, Sergey Zakharov, Dhimiter Bello

**Affiliations:** 1 Department of Occupational Medicine, First Faculty of Medicine, Charles University in Prague and General University Hospital in Prague Na Bojišti 1, 128 00 Prague 2, Czech Republic; Stepanka.Vlckova@vfn.cz (S.V.); zdenka.fenclova@lf1.cuni.cz (Z.F.); pavlina.klusackova@lf1.cuni.cz (P.K.); Sergej.Zacharov@vfn.cz (S.Z.); 2 Institute of Chemical Process Fundamentals of the CAS, Rozvojova 1/135, 165 02 Prague, Czech Republic; zdimal@icpf.cas.cz (V.Z.); ondracek@icpf.cas.cz (J.O.); schwarz@icpf.cas.cz (J.S.); kostejn@icpf.cas.cz (M.K.); 3 Institute of Biophysics and Informatics, First Faculty of Medicine, Charles University in Prague and General University Hospital in Prague, Salmovska 1, 120 00 Prague, Czech Republic; komarc@ftvs.cuni.cz; 4 Faculty of Physical Education and Sport, First Faculty of Medicine, Charles University in Prague and General University Hospital in Prague, José Marího 31, 162 52 Prague, Czech Republic; 5 Biocev, 1st Faculty of Medicine, Charles University, Prumyslova 595, 252 50 Vestec, Czech Republic; petr.kacer69@gmail.com; 6 Department of Machining and Assembly, Department of Engineering Technology, Department of Material Science, Faculty of Mechanical Engineering, Technical University in Liberec, 461 17 Liberec, Czech Republic; stepanka.dvorackova@tul.cz (S.D.); alespopov@yandex.ru (A.P.); 7 UMass, Lowell, Department of Biomedical and Nutritional Sciences, Zuckerberg College of Health Sciences, Lowell, MA 01854, USA; Dhimiter_Bello@uml.edu

**Keywords:** nanoparticles, nanocomposites, exhaled breath condensate (EBC), inflammation, spirometry, FeNO

## Abstract

Thousands of researchers and workers worldwide are employed in nanocomposites manufacturing, yet little is known about their respiratory health. Aerosol exposures were characterized using real time and integrated instruments. Aerosol mass concentration ranged from 0.120 mg/m^3^ to 1.840 mg/m^3^ during nanocomposite machining processes; median particle number concentration ranged from 4.8 × 10^4^ to 5.4 × 10^5^ particles/cm^3^. The proportion of nanoparticles varied by process from 40 to 95%. Twenty employees, working in nanocomposite materials research were examined pre-shift and post-shift using spirometry and fractional exhaled nitric oxide (FeNO) in parallel with 21 controls. Pro-inflammatory leukotrienes (LT) type B4, C4, D4, and E4; tumor necrosis factor (TNF); interleukins; and anti-inflammatory lipoxins (LXA4 and LXB4) were analyzed in their exhaled breath condensate (EBC). Chronic bronchitis was present in 20% of researchers, but not in controls. A significant decrease in forced expiratory volume in 1 s (FEV1) and FEV1/forced vital capacity (FVC) was found in researchers post-shift (*p* ˂ 0.05). Post-shift EBC samples were higher for TNF (*p* ˂ 0.001), LTB4 (*p* ˂ 0.001), and LTE4 (*p* ˂ 0.01) compared with controls. Nanocomposites production was associated with LTB4 (*p* ˂ 0.001), LTE4 (*p* ˂ 0.05), and TNF (*p* ˂ 0.001), in addition to pre-shift LTD4 and LXB4 (both *p* ˂ 0.05). Spirometry documented minor, but significant, post-shift lung impairment. TNF and LTB4 were the most robust markers of biological effects. Proper ventilation and respiratory protection are required during nanocomposites processing.

## 1. Introduction

Thousands of workers are engaged in the research, development, and commercial scale production of nano-enabled composites. Yet, limited data is available on exposures and more so on health effects, in occupational settings that produce and use nanomaterials [1,2,3] despite their frequent use in various industrial applications (e.g., construction, composites, fillers), with possible release during manufacturing [4,5,6,7].

Molecular epidemiological studies to date on workers handling engineered nanomaterials, suggest respiratory health impairment. Elevated pro-inflammatory markers, including-inflammatory cytokines, such as tumor necrosis factor (TNF) and interleukin (IL) 6 and IL 8 in the biological fluids (blood) of workers [8], elevated antioxidant enzymes and cardiovascular markers in circulation [9,10], higher pro-inflammatory leukotrienes (LTs) [11], and markers of oxidative stress in exhaled breath condensate (EBC) and/or circulation [12,13,14,15].

In the context of non-invasive sampling of the deep airways [16,17], EBC is one of the few means of non-invasive monitoring of individuals exposed to nanoparticles, and several lung injury biomarkers have been measured successfully and non-invasively in EBC of individuals exposed to nanoparticles. EBC is composed mainly of water (99.9%) and contains only a small proportion of water-soluble and insoluble compounds, which presumably originate from the airway lining fluid in the form of aerosolized particles generated during the re-opening of distal airways [18].

Several lung injury biomarkers have been measured successfully in EBC using highly sensitive liquid chromatography mass spectrometric (LC/MS) techniques. LTB4 is a potent inducer of chronic inflammation because of the activation of leukocytes. Cysteinyl LTs (LTC4, LTD4, and LTE4) contract airway smooth muscles and increase vascular permeability [19]. Proinflammatory LTs and anti-inflammatory lipoxins (LXs) are used as biomarkers of oxidative damage and lung fibrosis [19], including pneumoconiosis caused by silica and asbestos [20,21]. Under conditions of persistent oxidative damage, the homeostatic redox state of the subjects is perturbed, leading to an imbalance between the endogenous pro- and anti-inflammatory mediators in the lung. The pro-inflammatory process involves up-regulation of the transcription of various pro-inflammatory genes, including TNF, a monocyte-derived cytotoxin implicated in tumor regression, septic shock, cachexia, and several ILs, involved in humoral immunity, inflammation, and lung fibrosis [22].

In our previous studies, we report on elevated inflammatory and oxidative stress markers in EBC of workers exposed to aerosols containing about 80–85% nanoparticles of TiO_2_ [11,13] or Fe-oxides (Fe_2_O_3_) [23]. We have extended our previous investigation on lung injury in workers exposed to aerosols containing 80–85% nanoparticles of TiO_2_ or Fe-oxides to nanocomposite researchers and we have shown that nanocomposites workers have high markers of oxidative stress in EBC [24]. In this study of researchers involved in various steps of nanocomposite synthesis and processing during the research and development phase, we have expanded our repertoire of biomarkers to also include several pro-inflammatory cytokines/chemokines (TNF, IL 5, and IL 9), as well as anti-inflammatory IL 4, IL 10, and IL 13, and lipoxins LXA4 and LXB4 [25] in EBC. These cytokines/chemokines stimulate the growth, differentiation, and recruitment of mast cells, eosinophils, basophils, and B-cells, all of which are involved in humoral immunity and allergic responses.

We have used sensitive techniques such as liquid chromatography-electrospray ionization-tandem mass spectrometry (LC-ESI-MS/MS) or matrix-assisted laser desorption ionization time-of-flight (MALDI-TOF).

The primary aim of this work was to evaluate, for the first time, markers of inflammation in the EBC of workers exposed to nanoparticles during various tasks involved in the synthesis and post-processing of nanocomposite materials.

## 2. Materials and Methods

### 2.1. Workplace Processes

A detailed description of workplace processes was provided in a preceding paper [24]. The focus of this investigation was a research and development unit searching for thermoplastic or reactoplastic (thermoset) composite materials that exhibit new physical characteristics.

Researchers would normally work in two workshops: welding and smelting of mixtures containing nano-additives in workshop 1, and machining of the finished nanocomposite in workshop 2. All tasks occur simultaneously in both workshops and lasted on average 2.5 h. For simplicity, we refer to these examinations as pre-shift and post-shift, even though the remainder of their total 8 h shift was spent in their offices. Total duration of nanoparticles production in both workshops lasted about 4 h, each worker was exposed for about 2.5 h.

On the day of medical examination, in workshop 1, 11 researchers performed welding on metal surfaces on mild steel (content in wt%: Fe, 97.39; C, 0.24; Mn, 1.70; Si, 0.6; P, 0.035; S, 0.035). In the same workshop 1, an alloy (content in wt%: Al, 83.50; Si, 10.0; Fe, 0.8; Cu, 3.0; Mn, 0.55; Mg, 0.25; Cr, 0.15; Ni, 0.55; Zn, 1.2), mixed with modifying salts (NaCl, KCl, NaF), was smelted in the smelting oven at 760 ⁰C.

In workshop 2, machining of surfaces of nanocomposite blocks of geopolymers and epoxide resins with SiO_2_ fillers, (1.0% *w*/*w* nanoSiO_2_) was performed by the remaining nine researchers. Neither ventilation nor respiratory protection of any kind was used in both shops.

### 2.2. Workplace Aerosol Measurements

Real-time nanoaerosol monitoring was conducted with several aerosol spectrometers, including a scanning mobility particle sizer (TSI SMPS 3936L, Shoreview, MN, USA), and an aerodynamic particle sizer (TSI APS 3321, USA), covering the size range of aerosol particles from 6 nm up to 20 µm. Additionally, an ultrafine condensation particle counter (TSI UCPC 3025, TSI Inc., Shoreview, MN, USA), was used to measure the total particle number concentration (3 nm–1 µm), as well as three optical particle sizers (TSI OPS 3330, TSI Inc., Shoreview, MN, USA) were used to measure number size distribution in the range of 300 nm–10 µm.

The measurement of aerosols started 15 min before the beginning of the working tasks. In workshop 1, it lasted 95 min during welding and 100 min during smelting, in workshop 2, it lasted 120 min during machining. Total number of samples measured using SMPS/APS was 109.

Area and time integrated sampling was conducted using a Berner low pressure impactor (BLPI, HAUKE GmbH, Gmunden, Austria) to sample aerosol particles onto 10 stages corresponding to their aerodynamic diameter covering the 25 nm–13.6 µm size range [26].

Elemental analysis of size-resolved aerosol samples from the BLPI was performed using a scanning electron microscope equipped with energy-dispersive X-ray spectroscopy (SEM/EDX, XFlash detector 5010, Bruker, Karlsruhe, Germany).

### 2.3. Subjects

Pre-shift and post-shift EBC samples were collected in 20 nanocomposites researchers (15 men, 5 women; one smoker, 19 non-smokers; mean age 41.8 ± 11.4 years; exposure duration 17.8 ± 10.0 years). They were exposed for an average of 2.5 h in the workshops, the remainder of their total 8-h shift was spent in their offices. For simplicity, we refer to these examinations as pre-shift and post-shift, even though they were in fact post-exposure task measurements.

Similarly, 21 controls (15 men, 6 women; two smokers, 19 non-smokers; mean age 42.7 ± 11.5 years) working as office employees in the same town were examined.

First, they answered questions from a standardized questionnaire concerning their personal and occupational history. Chronic bronchitis was defined clinically as chronic, productive cough for three months in each of two successive years, and dyspnea was defined according to The New York Heart Association (NYHA).

All participants then underwent a physical examination, followed by collection of their EBC. Lastly, fractional exhaled nitric oxide (FeNO) and spirometry were also conducted. The researchers were examined twice, at first before exposure and then after the 2.5-h exposure. The controls were examined only once during the same time frame as the researchers.

The study was approved by the Ethical Committee of the Charles University according to the Helsinki criteria. All participants were informed of the study aim and signed an informed consent form before the study began.

FeNO was measured by a portable Hypair FeNO analyzer (Medisoft, Belgium). According to ATS/ERS recommendations, a FeNO result greater than 50 ppb was considered elevated, whereas values 25–50 ppb were considered borderline [27].

Spirometry was performed by a SpiroPro, Jaeger, Germany. The measurement included forced vital capacity (FVC), inspiratory vital capacity (VCIN), peak expiratory flow (PEF), and forced expiratory volume in 1 s (FEV1). These parameters were considered lower than normal if they were less than 80% of the predicted values (i.e., comparing with the population with similar characteristics, such as age, sex, height, and weight), and if the FEV1/FVC ratio was less than 0.75.

### 2.4. Collection and Analysis of Inflammation Markers in EBC

EBC samples were collected using Ecoscreen Turbo (DECCS, Jaeger, Germany). All subjects breathed tidally for about 15 min through a mouthpiece connected to a condenser (−20 °C) while wearing a nose-clip. A minimum exhaled air volume of 120 L was maintained through the EcoVent device (Jaeger, Wurzburg, Germany). All samples were immediately spiked with deuterium labelled standards, immediately frozen and stored at −80 °C for subsequent processing.

Analyses of low-molecular biomarkers LTs and LXs were performed using LC-ESI-MS/MS, consisting of a quaternary pump, Accela 600, and Accela autosampler coupled with a triple quadrupole mass spectrometer TSQ Vantage, equipped with heated electrospray ionization (HESI) (Thermo Fisher Scientific, Waltham, MA, USA). Then, solid-phase extraction (SPE) for rapid and effective isolation of biomarkers from the EBC, and MS/MS detection were used, as previously described [28].

ILs and TNF were analyzed by a matrix-assisted laser desorption ionization time-of-flight (MALDI-TOF) using an Autoflex mass spectrometer (Bruker Daltonics, Bremen, Germany) with a MALDI sample target (600 µm Chip™; Bruker Daltonics, Bremen, Germany) [28,29]. To exclude contamination of EBC by saliva, α-amylase concentration was determined and the pH of EBC was measured [30].

### 2.5. Statistical Analysis

Basic descriptive statistics (mean, median, confidence interval, standard deviation, skewness, and kurtosis) were computed, which were subsequently tested for normality using the Kolmogorov–Smirnov test. To compare frequency counts of demographic categorical variables (e.g., smoking, alcohol consumption) in groups of workers versus controls, the Fischer exact test was used. Differences in interval variables (e.g., spirometry parameters, markers of inflammation in EBC, FeNO) were tested using the Mann–Whitney U test (for non-normally distributed variables, i.e., spirometry parameters, markers of inflammation in EBC, FeNO). The paired sample t-test (or the Wilcoxon signed-rank test) was used to compare workers’ pre-shift and post-shift values of the markers of inflammation. The bivariate relationship between variables under study was assessed using the Spearman correlation coefficient. Multiple regression analysis was used to predict markers in EBC by a set of predictors. Two sets of regression models were specified: (a) for controls and pre-shift data (merged, N = 41), and (b) for controls and post-shift data (merged, N = 41). Statistical significance was set at *p* < 0.05. All analyses were conducted using SPSS version 22.0 (SPSS, Inc., Chicago, IL, USA).

## 3. Results

### 3.1. Workplace Aerosol Measurements

Exposure data have been provided in detail in a preceding manuscript [24] and have been briefly summarized here for completeness. Mean total mass concentration in the workshops obtained from BLPI was 0.120 mg/m^3^ during smelting, 0.804 mg/m^3^ during machining, and 1.840 mg/m^3^ during welding. Total median particle number concentration was 1.3 × 10^5^ particles/cm^3^ (#/cm^3^) and the interquartile range (IQR) was from 1.2 × 10^5^ to 1.5 × 10^5^ #/cm^3^ during welding, 4.8 × 10^4^ #/cm^3^ (IQR 3.1 × 10^4^ to 9.0 × 10^4^ #/cm^3^) during smelting, and 5.4 × 10^5^ #/cm^3^ (IQR 3.1 × 10^5^ to 6.8 × 10^5^ #/cm^3^) during machining. The highest proportion of particles smaller than 100 nm in diameter was found during smelting, at 95%, followed by machining (61%), and welding (40%). Chemical analysis of airborne aerosols for all three operations revealed the following elements presented in their descending order of abundance: Fe, Mn, Si, Na, S, Cl, Al, Ca, K, Mg, and Ti. Chemical analysis of the nano-sized fraction showed prevailing Fe, Mn, Si, Al, S, Cl, and K [24].

### 3.2. Subjects Characteristics

The group characteristics did not differ significantly, including smoking (5% smokers among workers with 10 pack-years; 9.5% in controls with average 14 pack-years) and daily alcohol consumption (90% in workers and 85% in controls). No difference was seen between the characteristics of the subgroups of researchers working in workshop 1 and workshop 2.

### 3.3. Respiratory Disease/Symptoms

Dyspnea, associated with minor limitations in ordinary physical activity, and chronic bronchitis were found only in exposed subjects (Table 1). Cough was five times more frequent than in the controls. The only exposed smoker did not present symptoms of chronic bronchitis, neither did the two smokers from the control group.

### 3.4. Spirometry

Lung function parameters were within the normal range in both researchers and controls. However, the post-shift FEV1, %FEV1, and FEV1/FVC ratio declined significantly in researchers relative to the pre-shift measurements; the changes were modest (Table 2B). Duration of employment (in years) as a researcher in nanocomposites was associated with a decline in the post-shift FEV1/FVC ratio.

### 3.5. FeNO

**Figure 1:**
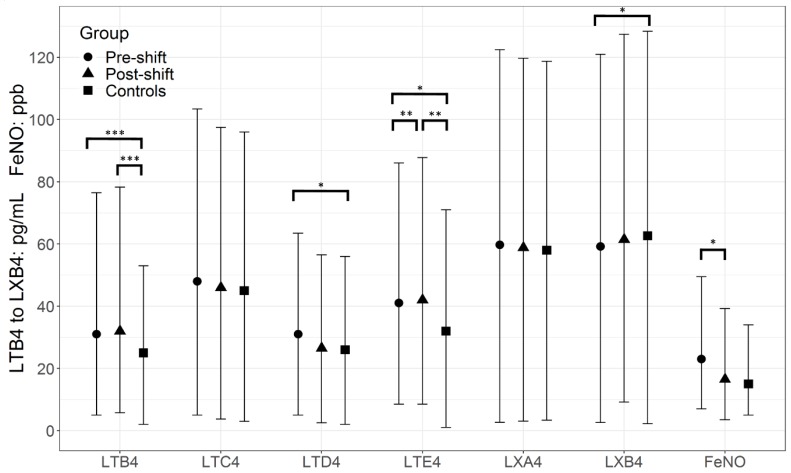
Comparison of pre- and post-shift levels (median ± interquartile range) of inflammatory markers leukotrienes (LT) (LTB4, LTC4, LTD4, LTE4), anti-inflammatory markers lipoxins (LX) (LXA4, LXB4), and fractional exhaled nitric oxide (FeNO) in the exhaled breath condensate of 20 nanocomposite synthesis workers relative to 20 controls. * (*p* < 0.05) ** (*p* < 0.01) *** (*p* < 0.001).

Median and mean FeNO levels in researchers did not differ significantly from the controls (Figure 1 and Figure S1) and post-shift FeNO was lower than the pre-shift level. No association was found between FeNO, respiratory symptoms, and markers of inflammation in the EBC.

### 3.6. Markers in EBC

**Figure 2:**
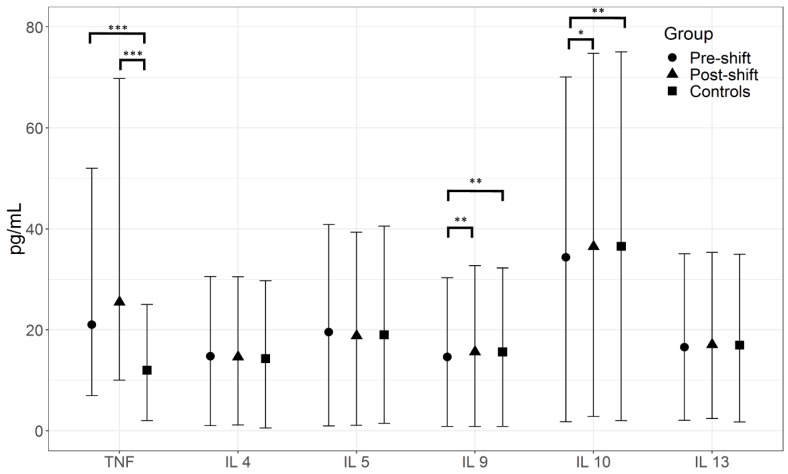
Comparison of pre- and post-shift levels (median ± interquartile range) of pro-inflammatory cytokines tumor necrosis factor (TNF); interleukins (IL) IL-5, IL-9; and anti-inflammatory IL-4, IL-10, and IL-13 in the exhaled breath condensate of 20 nanocomposite synthesis workers compared with 21 controls. * (*p* < 0.05) ** (*p* < 0.01) *** (*p* < 0.001).

Several pre-shift and post-shift pro-inflammatory markers were elevated relative to controls as shown in Figure 1 and Figure 2 (medians), and Figures S1 and S2 (means). This includes LTB4, LTD4, LTE4, as well as TNF. Not surprisingly, the anti-inflammatory markers, LXB4, and IL 10 were lower in the researchers relative to controls. No statistically significant difference was seen between EBC markers of the subgroup of researchers working in workshop 1 and workshop 2.

For some pro-inflammatory markers, most notably LTE4, IL 9, and IL 10, the post-shift values were higher, whereas for other markers, either there were no differences or no clear trends.

There was no significant difference in pH of EBC samples of both groups. Amylase concentrations were less than 0.01% of those in saliva in all samples.

Duration of employment (years) as a nanocomposite researcher correlated positively with pre-shift TNF and negatively with post-shift anti-inflammatory LXB4. Allergic rhinitis, cough, and chronic bronchitis in the exposed subjects correlated with lower pH in both pre- and post-shift EBC samples, as presented in Table S1. No markers correlated with smoking.

The levels of several markers in the pre-shift and the post-shift samples correlated, for example, with pH, LTs, FeNO, and IL 4.

In addition, some correlations between different pre-shift and post-shift markers were found. They are shown in Table S2 for LTB4 and TNF, which were the markers with the highest statistical significance. Anti-inflammatory marker LXB4 showed negative correlations with other biomarkers.

### 3.7. Multiple Regression Analysis

Confirmed significant association between nanocomposites handling and pre-shift and post-shift LTB4, LTE4, and TNF, as shown in Table 3.

Markers of oxidative stress were presented in an accompanying manuscript [24]. Here, we explore their relationship with inflammatory markers. A total of 23 correlations were observed between oxidative stress markers (of lipids, protein, and nucleic base pairs) and inflammatory markers, especially for pre-shift LTB4 and TNF, as shown in Table S3.

## 4. Discussion

A large body of in vivo and in vitro nanotoxicology studies have shown that nanoparticles induce intracellular reactive oxygen species, and pro-inflammatory mediators [16,31]. Much less data are available concerning studies on workers handling nanomaterials, where exposures are complex and highly variable.

This study deals with long-term employed subjects in nanocomposites research, where the proportion of nanoparticles in the aerosol during handling of nanocomposites ranged from 40% to 95%. The chemical analysis of the nano-sized fraction in these processes identified predominantly Fe, Mn, Al, S, and Si [24]. High concentrations of nano-aerosols are common during heating of metal at high temperatures, welding, grinding, and machining of a variety of other nanocomposite materials [32,33]. Despite this well documented exposure scenario, the researchers were not using any respiratory protection. As a result, chronic short-term exposures to mixtures containing a substantial fraction of nanoparticles for 2.5 h/day were sufficient at inducing respiratory effects at both the molecular level (elevated markers of inflammation and oxidative stress pre- and post-shift), as well as higher vulnerability at organ function level, as measured by post-shift spirometry (minor, but significant reduction of FEV1, %FEV1, and FEV1/FVC ratio), and 20% chronic bronchitis. This is one of the few occupational nanoparticle exposure studies to show respiratory effects from such short-duration daily exposures.

In our earlier studies, LTs were elevated in TiO_2_ exposed workers, relative to controls [11]. LTB4 only, but not cysteinyl LTs, was elevated in workers exposed to nano Fe-oxides [23].

In this study of nanocomposite manufacturing workers, we did find elevated LTB4 and LTE4, but not LTC4 and post-shift LTD4. These differences may reflect different exposure profiles, including exposure intensities, size distributions, chemical composition, as well as frequency and duration of tasks. For example, exposure duration was on average 4 h in TiO_2_ production workers and 2.5 h in nanocomposite workers.

LTB4 levels in nanocomposite research workers in this study were ~30% lower than in workers exposed to nanoTiO_2_ (mean 53 and 49 pg/mL in 2012 and 2013, respectively), but 40% higher than LTB4 in the EBC of office workers of the same factory who spent about 15 min daily in the workshops (28 pg/mL) [11].

Both pre-shift and post-shift TNF overexpression were positively associated with exposure, as well as with several other oxidative stress and inflammatory markers in other human studies [8,33,34]. TNF seems to be an important inflammatory biomarker as it was correlated with the length of the employment in the nanocomposites researchers and with the decline in the FEV1/FVC ratio. In researchers exposed to nanocomposites, chronic bronchitis appeared more frequent relative to controls and we have found a small but significant decrease in %FEV1 and FEV1/FVC post-shift, pointing to lung obstruction. Three exposed subjects complained of light dyspnea, but nobody from the controls described this symptom. In addition, dyspnea correlated with lower EBC pH (*p* = 0.039).

Both pro-inflammatory cytokines/chemokines (IL 5 and IL 9), and anti-inflammatory IL 4, IL 10, IL 13, LXA4, and LXB4 were examined.

The anti-inflammatory eicosanoids mediators that are derived from the arachidonic acid are involved in the resolution of acute inflammatory responses and initiate tissue repair in response to injury, infection, or allergy [35,36,37]. LXB4 and IL 10 are anti-inflammatory biomolecules that play an important role in resolution of inflammation. In responses to acute exposures, these biomarkers tend to go up with other pro-inflammatory markers and then go down, as the inflammation is resolved. In chronic exposures with established inflammation, the anti-inflammatory biomarkers, such as IL 10, are not playing an important role, presumably because the feedback loops that require them are no longer functional [38,39]. In this study, LXB4 and IL 10 were lower in the pre-shift samples, compared with controls, which may reflect a reduced response due to long-term exposures. Post-shift increases of their concentration may reflected only minor temporal variations in response to this exposure, but overall, their concentrations did not reach biologically significant levels, as compared with controls.

We have confirmed that these mediators end up in the EBC and we could see several negative correlations of the anti-inflammatory LXB4 with the pro-inflammatory markers, especially TNF and with several markers of oxidative stress [24].

Pro-inflammatory IL 9 is an important player in the pathogenesis of bronchial hyperresponsiveness that affects various cell types involved in immunity and inflammation [22]. Again, elevation of post-shift concentrations of IL 9 is indicative that nanoparticle exposures in nanocomposite workers can trigger Th-mediated responses and induce airway hyper-responsiveness as documented by the presence of chronic bronchitis and changes in %FEV1 and FEV1/FVC. The changes in ILs and LXs were only minor and their relationships with exposure were not robust, making these markers less helpful.

FeNO, which decreased during the shift, does not seem useful, accordingly to both our nanoTiO_2_ studies [11] and the study of Glass et al. [40]. This agrees with clinical data found in exposed individual researchers (chronic bronchitis, but no asthma or rhinitis). Several mechanisms have been suggested for lower FeNO, for instance, a predominantly increased neutrophilic inflammation in the lungs, rather than allergic eosinophilic pulmonary inflammation [41]. Another, more plausible explanation is that oxidative processes in the airway mucosa induced by nanoparticles may lead to the direct consumption or scavenging of NO, similarly to cigarette smoke exposure [42].

Our findings are consistent with limited epidemiological studies in nanomanufacturing workers, in which reduction of lung function parameters and an increase of TNF or oxidative stress markers were described [15,43,44]. In copier operators, exposed predominantly to a mixed-type exposure to copier emitted nanoparticles that contain a few percent (1–8%) metal oxides, respiratory, immunological, cardiovascular, and other disorders may develop following nanoparticle inhalation [38,39,45]. A decade into nanomanufacturing, we are now starting to see the emergence of disease in these workers, especially respiratory airway disorders.

In addition, markers of oxidation of lipids, nucleic acids, and proteins used in our studies were so sensitive that they could distinguish between industrial production workers exposed to TiO_2_, as well as their respective office workers attending the workshops only for a brief period of time [13,46,47].

It is important to mention that the main source of airborne nanoparticles in the workshops originated from manufacturing and post-processing of nanocomposites, including smelting, welding, possibly handling of nano- powders (which was not monitored here), and mechanical processing such as trimming and polishing. As such, these nanoparticles are of mixed origin (incidental and engineered) and mixed composition. It is not possible, and perhaps not advisable, to try and parse out the relative contribution of such nanoparticles on the health outcomes. On the contrary, such research settings should adopt better exposure controls and increase researchers’ awareness about such exposures and their possible health effects. In occupational cohorts exposed to nanoparticles and other particles in general, respiratory disorders typically develop under chronic exposures over many years to decades [48]. Predicting future disease progression from earlier markers of tissue injury/damage, albeit highly desirable, remains, at present, a major obstacle.

The main limitation of this study is the relatively low number of exposed subjects. Unfortunately, the research and development nature of the nanocomposite sector (and nanomanufacturing in general) is characterized by small and unstable occupational cohorts of workers. As a result, the number of similar studies is also remarkably limited.

Another limitation is that we could not measure personal exposures and link exposures with airway dosimetry. A one-day exposure characterization is not representative of long-term exposures to these workers. Therefore, we plan to continue to monitor them over time and generate personal exposure data and better exposure summaries.

## 5. Conclusions

This study provides evidence that occupational exposure to inhaled nanomaterials generated during various tasks associated with nanocomposite synthesis, production, and post-processing (in both workshops) causes respiratory impairments and subclinical spirometry changes. Several markers of inflammations were elevated both pre- and post-shift, with an underlying chronic neutrophilic, not eosinophilic, inflammation, according to FeNO results and clinical findings. The length of the employment in the nanocomposites production correlated with TNF and a decline in the FEV1/FVC ratio, which supports the chronic effect of exposure to nanomaterials. These findings and those from our earlier studies provide evidence in favor of the usefulness of monitoring inflammatory markers, such as TNF, in EBC. Overexpression of TNF in EBC complements our earlier findings of DNA, protein, and lipid damage in the EBC of workers with nanoTiO_2_ and nano Fe-oxides, and even in this group of nanocomposites research workers. Better exposure controls and increased awareness are needed in this as well as similar nanomanufacturing sectors.

## Figures and Tables

**Table 1:** Respiratory symptoms and disorders in the groups of subjects.

Symptom/Disorder	Exposed	Controls	
N	20	21	*p* Value
**Acute bronchitis/bronchopneumonia in past five years (n, %)**	10 (50.0%)	9 (42.9%)	0.758
**Allergic rhinitis (n, %)**	7 (35.0%)	10 (60.0%)	0.530
**Asthma (n, %)**	1 (5.0%)	1 (4.8%)	1.000
**Cough (n, %)**	5 (25.0%)	1 (4.8%)	0.093
**Chronic bronchitis (n, %)**	4 (20.0%)	0/0%	*
**Dyspnea (NYHA class II) (n, %)**	3 (15.0%)	0 (0%)	*

Dyspnea NYHA class II = slight limitation of physical activity according to New York Heart Association (NYHA) Functional Classification. * *p*-values excluded from comparisons due to 0 prevalence in the controls.

**Table 2:** Lung functions in the workers pre-shift and post-shift and in the controls.

Examination	FVC (L)	%FVC	VCIN (L)	%VCIN	FEV1 (L)	%FEV1	FEV1/FVC	PEF (L/min)	%PEF
Pre-shift	4.33 ± 1.02	94.70 ± 13.30	4.36 ± 1.01	92.15 ± 13.04	3.86 ± 0.95	102.20 ± 13.54	0.89 ± 0.06	9.77 ± 1.95	110.15 ± 14.28
Post-shift	4.33 ± 0.91	94.95 ± 11.64	4.39 ± 0.90	93.05 ± 11.01	**3.73 ± 0.81 ^⁰^**	99.00 ± 12.03 *	**0.86 ± 0.06 ^+^**	9.44 ± 2.06	106.80 ± 15.19
Controls	4.43 ± 1.05	100.76 ± 13.63	4.47 ± 1.08	98.71 ± 13.04	3.88 ± 0.96	106.10 ± 13.96	0.89 ± 0.06	9.72 ± 1.80	111.81 ± 20.17

FVC = forced vital capacity, FEV1 = forced expiratory volume in 1 s, VCIN = inspiratory vital capacity, PEF = peak expiratory flow, % = of predictive value for the subjects of similar characteristics (age, sex, height, and weight). **^⁰^** comparison with the pre-shift level, *p* = 0.019; * comparison with the pre-shift level, *p* = 0.011; **^+^** comparison with the pre-shift level, *p* = 0.031; bold numbers show significant differences

**Table 3:** Multiple regression analysis between biomarkers in exhaled breath condensate in nanocomposites production workers (regression coefficient and 95% confidence interval (CI)) and several independent variables (age, gender, alcohol, and body mass index (BMI)). Two sets of regression models were specified: (a) for controls and pre-shift data (merged, N = 41), and (b) for controls and post-shift data (merged, N = 41).

	Pre-Shift					Post-Shift		
Markers	LTB4	LTD4	LTE4	LXB4	TNF	LTB4	LTE4	TNF
**Nanocomposites production** **(Yes/No)**	10.70 *** (4.89, 16.51)	3.09 * (0.24, 5.95)	4.35 * (0.19, 8.51)	−3.23 * (−6.01, −0.45)	10.58 *** (6.21, 14.95)	10.51 *** (4.85, 16.17)	5.22 * (0.94, 9.50)	20.34 *** (11.07, 29.61)
**Age (years)**	0.31 * (0.03, 0.58)	−0.08 (−0.21, 0.06)	−0.05 (−0.25, 0.15)	0.05 (−0.08, −0.18)	0.20 (−0.01, 0.41)	0.29 * (0.02, 0.56)	−0.06 (−0.26, 0.14)	−0.06 (−0.50, 0.39)
**Gender (Male/Female)**	4.14 (−3.21, 11.48)	−0.57 (−4.17, 3.04)	−4.72 (−9.97, 0.54)	−0.25 (−3.72, 3.21)	2.56 (−2.97, 8.08)	3.92 (−3.23, 11.08)	−5.01 (−10.42, 0.40)	7.61 (−4.12, 19.33)
**Alcohol (Yes/No)**	9.41 (−0.29, 19.10)	1.17 (−3.59, 5.93)	0.68 (−6.26, 7.62)	−2.35 (−6.93, 2.22)	5.73 (−1.57, 13.03)	8.57 (−0.87, 18.01)	0.01 (−7.14, 7.16)	1.09 (−14.39, 16.57)
**BMI (kg/m^2^)**	−0.07 (−0.65, 0.51)	0.15 (−0.14, 0.44)	0.18 (−0.23, 0.60)	0.02 (−0.26, 0.29)	−0.16 (−0.60, 0.28)	−0.08 (−0.65, 0.48)	0.19 (−0.24, 0.61)	−0.36 (−1.29, 0.56)

*** (*p* < 0.001), * (*p* < 0.05). LT—leukotrienes; LX—lipoxin; TNF—tumor necrosis factor; bold numbers show significant differences.

## Supplementary Materials

The following are available online at http://www.mdpi.com/2079-4991/8/9/731/s1. Figure S1: Comparison of mean pre- and post-shift levels of inflammatory markers leukotrienes (LT) LTB4, LTC4, LTD4, LTE4), anti-inflammatory markers lipoxins (LX) (LXA4, LXB4), and fractional exhaled nitric oxide (FeNO) in the exhaled breath condensate of 20 nanocomposite synthesis workers relative to 21 controls. * (*p* < 0.05) ** (*p* < 0.01) *** (*p* < 0.001). Figure S2: Comparison of mean pre- and post-shift levels of pro-inflammatory cytokines tumor necrosis factor (TNF); interleukins (IL) 5, IL 9; and anti-inflammatory IL 4, IL 10, and IL 13 in the exhaled breath condensate of 20 nanocomposite synthesis workers compared with 21 controls. * (*p* < 0.05) ** (*p* < 0.01) *** (*p* < 0.001). Table S1 Correlations of pre-shift and post-shift EBC markers with selected characteristics, exposure parameters and respiratory symptoms in the workers exposed to nanocomposites. Table S2 Correlations of the pre-shift (1-) and post-shift (2-) inflammation markers leukotriene (LT) B4 and tumor necrosis factor (TNF) in the exhaled breath condensate (EBC) of the workers with inflammation markers LTC4, fractional exhaled nitric oxide (FeNO), and anti-inflammatory lipoxins (LXA4, LXB4). Table S3 Correlations between the markers of inflammation and markers of oxidative stress in the exhaled breath condensate of the workers.
