# Supplementary material for: Deep Airway Inflammation and Respiratory Disorders in Nanocomposite Workers
Source: Nanomaterials (Basel). 2018 Sep 16;8(9):731. doi: 10.3390/nano8090731 (PMC6164906; doi:10.3390/nano8090731)
Supplement: Supplementary file 1 [file nanomaterials-08-00731-s001.pdf]

Figure S1. Comparison of mean pre- and post-shift levels of inflammatory markers leukotrienes (LT) LTB4, LTC4, LTD4, LTE4), anti-inflammatory markers lipoxins (LX) (LXA4, LXB4), and fractional exhaled nitric oxide (FeNO) in the exhaled breath condensate of 20 nanocomposite synthesis workers relative to 21 controls. *(p<0.05) **(p<0.01) *** (p<0.001).

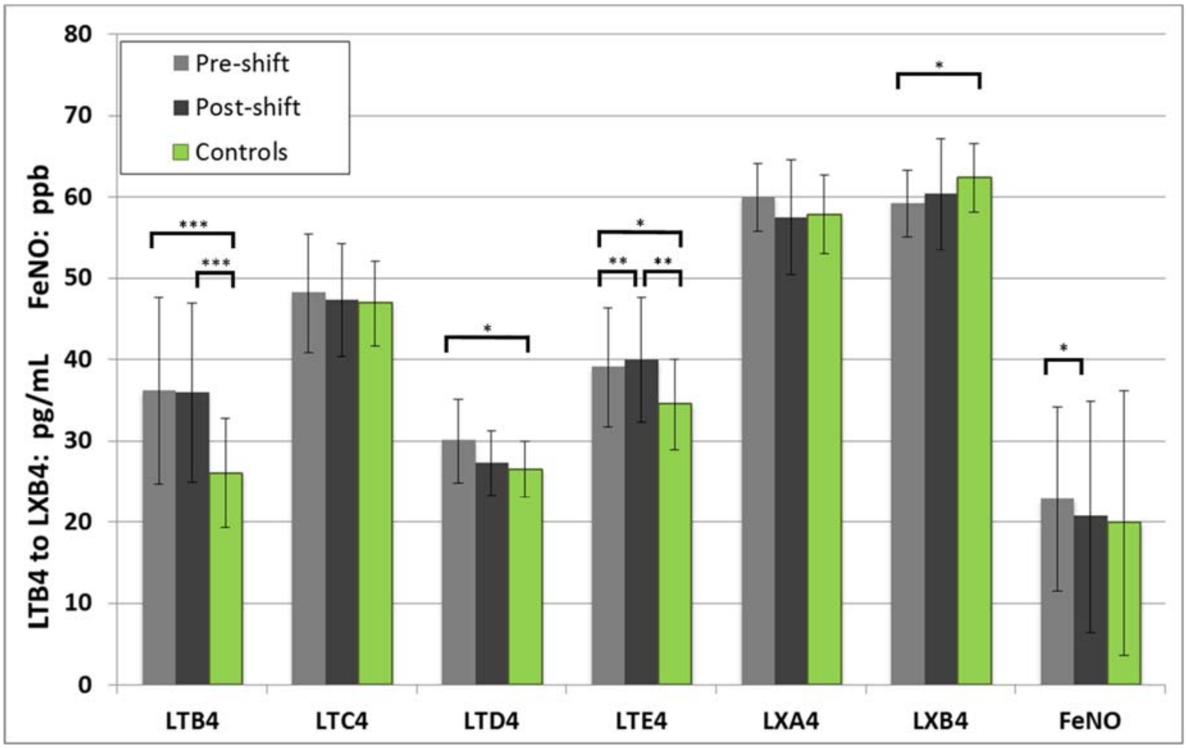

Figure S2: Comparison of mean pre- and post-shift levels of pro-inflammatory cytokines tumor necrosis factor (TNF), interleukins (IL) IL 5, IL 9 and anti-inflammatory IL 4, IL 10, and IL 13 in the exhaled breath condensate of 20 nanocomposite synthesis workers compared with 21 controls. *(p<0.05) **(p<0.01) *** (p<0.001)

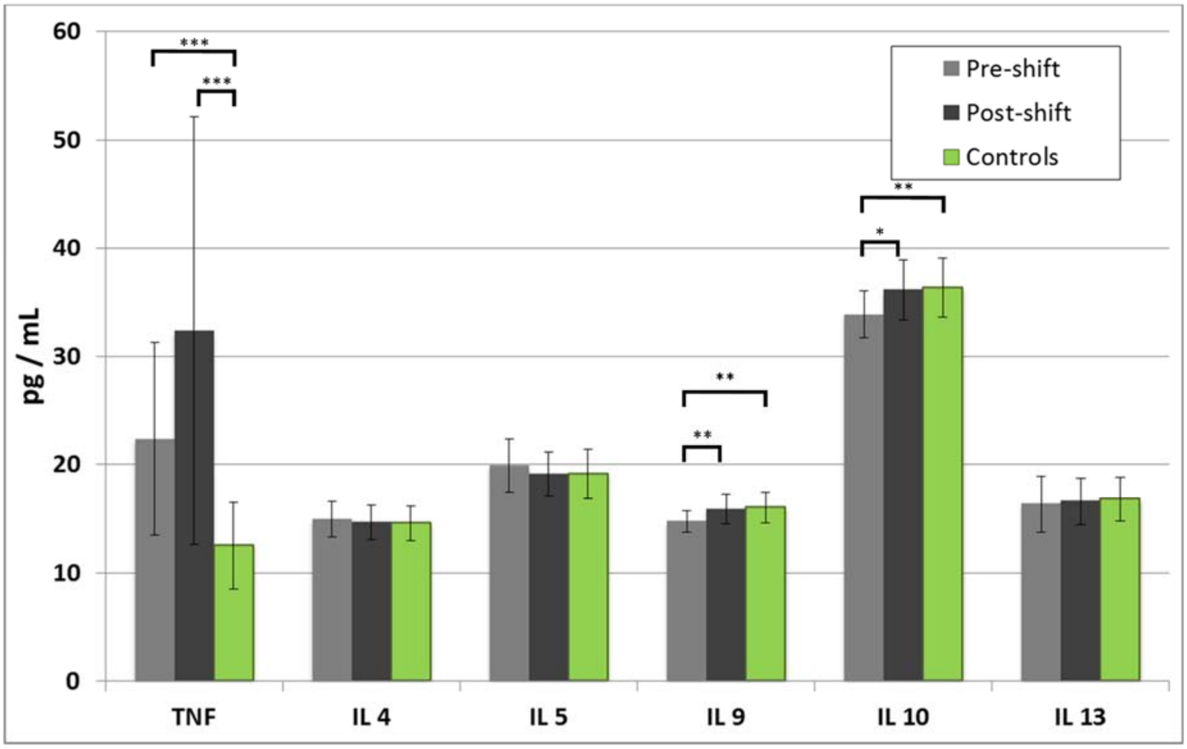

Table S1 Correlations of pre-shift and post-shift EBC markers with selected characteristics, exposure parameters and respiratory symptoms in the workers exposed to nanocomposites.

Characteristics	Pre-shift marker, correlation, p	Post-shift marker, correlation, p
**Employment in nanocomposites production (years)**	TNF, 0.473 (0.035)	LXB4, -0.449 (0.047)
**Acute bronchitis/ bronchopneumonia in past five years**	LTB4, 0.451 (0.046)<br>LTC4, 0.503 (0.024)<br>LXB4, -0.451 (0.046)	LTB4, 0.451 (0.046)<br>LTC4, 0.502 (0.024)
**Allergic rhinitis**	pH, -0.455 (0.044)	pH, -0.483 (0.031)
**Chronic bronchitis**	pH, -0.467 (0.038)	pH, -0.543 (0.013)
**Cough**	pH, -0.481 (0.032)	pH, -0.582 (0.007)
**Dyspnea (NYHA class II)**	LTC4, 0.465 (0.039)	LTC4, 0.464 (0.039)

Table S2: Correlations of the pre-shift (1-) and post-shift (2-) inflammation markers leukotriene (LT) B4 and tumor necrosis factor (TNF) in the exhaled breath condensate (EBC) of the workers with inflammation markers LTC4, fractional exhaled nitric oxide (FeNO), and anti-inflammatory lipoxins (LXA4, LXB4).

**Marker 1**	**Marker 2**	**Correlation Coefficient**	**Sig. (2-tailed)**
**LTB4**	**Other Marker**		
1-LTB4	1-LTC4	0.571	0.009
1-LTB4	1-LXB4	-0.472	0.036
1-LTB4	1-TNF	0.781	0.000
1-LTB4	2-LTC4	0.474	0.035
2-LTB4	1-LTB4	0.976	0.000
2-LTB4	1-LTC4	0.537	0.015
2-LTB4	1-LXB4	-0.462	0.040
2-LTB4	1-TNF	0.771	0.000
**TNF**	**Other Marker**		
1-TNF	1-LTB4	0.781	0.000
1-TNF	1-LTC4	0.627	0.003
1-TNF	1-LXB4	-0.573	0.008
1-TNF	2-LTB4	0.771	0.000
1-TNF	2-LTC4	0.516	0.020
2-TNF	2 FeNO	0.545	0.013
2-TNF	2-IL 9	-0.532	0.016

Table S3: Correlations between the markers of inflammation and markers of oxidative stress in the exhaled breath condensate of the workers.

**Pre-shift**	**MDA**	**HNE**	**HHE**	**C6-C13**	**8-iso**	**8-OHdG**	**8-OHG**	**5-OHMeU**	**o-Tyr**	**3-ClTyr**	**3-NOTyr**
**LTB4**	-0.359	0.013	0.056	0.111	0.039	**0.632**[b]	**0.586**[b]	**0.640**[b]	**0.776**[c]	**0.637**[b]	-0.028
**LTC4**	-0.030	0.123	0.183	0.317	0.066	0.414	0.402	0.290	0.217	**0.558**[b]	0.077
**LTD4**	0.382	0.150	**0.501**[a]	**0.485**[a]	0.129	0.268	0.223	-0.203	-0.231	0.079	-0.307
LXB4	*0.103*	*-0.101*	*-0.416*	*-0.361*	*-0.365*	***-0.698***[c]	***-0.480***[a]	*-0.398*	*-0.345*	*-0.383*	*0.067*
**TNF**	**-0.480**[a]	0.290	0.204	0.161	-0.066	**0.707**[c]	**0.503**[a]	**0.790**[c]	**0.577**[b]	**0.781**[c]	0.142
IL 4	*-0.376*	*0.064*	*-0.078*	***-0.496***[a]	*-0.169*	*0.172*	*0.005*	*0.106*	*-0.044*	*0.178*	*0.108*
**IL 5**	0.235	-0.201	0.356	**0.478**[a]	0.236	0.340	0.291	0.041	0.248	-0.032	-0.384
**Post-shift**	**MDA**	**HNE**	**HHE**	**C6-C13**	**8-iso**	**8-OHdG**	**8-OHG**	**5-OHMeU**	**o-Tyr**	**3-ClTyr**	**3-NOTyr**
**LTB4**	-0.193	0.032	0.050	0.175	0.214	0.332	**0.611**[b]	0.401	**0.792**[c]	**0.667**[b]	-0.064
**LTC4**	-0.063	0.123	0.091	0.369	0.094	0.139	0.241	0.142	0.134	**0.473**[a]	0.097
LXA4	*-0.112*	*-0.140*	*-0.206*	*0.338*	***0.462***[a]	*0.095*	*0.131*	*0.341*	*0.246*	*0.089*	*-0.189*

**FeNO**	0.098	0.173	-0.041	0.040	-0.046	0.280	0.245	**0.458**[a]	0.307	0.258	-0.392
**IL5**	-0.139	-0.170	-0.074	-0.128	0.278	-0.197	0.089	-0.031	0.378	0.215	**0.448**[a]

Significant correlations are in bold, [a] ($p < 0.05$), [b] ($p < 0.01$), [c] ($p < 0.001$); anti-inflammatory markers (LX, IL 4) are in italics.

LT=leukotriene, LX= lipoxin, TNF= tumor necrosis factor, IL= interleukin, FeNO= fractional exhaled nitric oxide, MDA=malondialdehyde, HNE=4-hydroxy-trans-nonenal, HHE=4-hydroxy-trans-hexenal, C6-13 =aldehydes C6-C13, 8-iso=8-isoProstaglandin F2α, 8-OHdG=8-hydroxy-2-deoxyguanosine, 8-OHG=8-hydroxyguanosine, 5-OHMeU=5-hydroxymethyl uracil, o-Tyr=o-tyrosine, 3-ClTyr= 3-NOTyr=3-nitrotyrosine.
